# Flu-IV score: a predictive tool for assessing the risk of invasive mechanical ventilation in patients with influenza-related pneumonia

**DOI:** 10.1186/s12890-022-01833-2

**Published:** 2022-01-29

**Authors:** Liang Chen, Xiudi Han, YanLi Li, Chunxiao Zhang, Xiqian Xing

**Affiliations:** 1 Department of Infectious Diseases, Nanjing Lishui People’s Hospital, No. 86 Chongwen Road, Lishui District, Nanjing, China; 2 Department of Infectious Diseases, Beijing Jishuitan Hospital, 4th Medical College of Peking University, Beijing, China; 3 Department of Pulmonary and Critical Care Medicine, Qingdao Municipal Hospital, Qingdao City, Shandong Province China; 4 Department of Infectious Diseases and Clinical Microbiology, Beijing Chao-Yang Hospital, Capital Medical University, Beijing, China; 5 Department of Pulmonary and Critical Care Medicine, Beijing Huimin Hospital, Beijing, China; 6 Department of Pulmonary and Critical Care Medicine, The 2nd People’s Hospital of Yunnan Province, Kunming City, Yunnan Province China

**Keywords:** Influenza, Pneumonia, Invasive mechanical ventilation, Prediction rule

## Abstract

**Background:**

The need for invasive mechanical ventilation (IMV) is linked to significant morbidity and mortality in patients with influenza-related pneumonia (Flu-p). We aimed to develop an assessment tool to predict IMV among Flu-p patients within 14 days of admission.

**Methods:**

In total, 1107 Flu-p patients from five teaching hospitals were retrospectively enrolled from January 2012 to December 2019, including 895 patients in the derivation cohort and 212 patients in the validation cohort. The predictive model was established based on independent risk factors for IMV in the Flu-p patients from the derivation cohort.

**Results:**

Overall, 10.6% (117/1107) of patients underwent IMV within 14 days of admission. Multivariate regression analyses revealed that the following factors were associated with IMV: early neuraminidase inhibitor use (− 3 points), lymphocytes < 0.8 × 10^9^/L (1 point), multi-lobar infiltrates (1 point), systemic corticosteroid use (1 point), age ≥ 65 years old (1 points), PaO_2_/FiO_2_ < 300 mmHg (2 points), respiratory rate ≥ 30 breaths/min (3 points), and arterial PH < 7.35 (4 points). A total score of five points was used to identify patients at risk of IMV. This model had a sensitivity of 85.5%, a specificity of 88.8%, and exhibited better predictive performance than the ROX index (AUROC = 0.909 vs. 0.594, *p* = 0.004), modified ROX index (AUROC = 0.909 vs. 0.633, *p* = 0.012), and HACOR scale (AUROC = 0.909 vs. 0.622, *p* < 0.001) using the validation cohort.

**Conclusions:**

Flu-IV score is a valuable prediction rule for 14-day IMV rates in Flu-p patients. However, it should be validated in a prospective study before implementation.

**Supplementary Information:**

The online version contains supplementary material available at 10.1186/s12890-022-01833-2.

## Background

Influenza is a common viral respiratory disease that affects between 5 and 10% of the population of the world each year, resulting in 3–5 million severe infections and between 290,000 and 650,000 annual deaths attributable to influenza-related illness [1]. Owing to the significant morbidity and mortality associated with disease, influenza is considered by many researchers to be one of the greatest threats to global public health [2]. So, even during the pandemic of COVID-19, the prevention and treatment of influenza should not be ignored.

Influenza-related pneumonia (Flu-p) is a severe form of influenza infection associated with over 50% of influenza-related hospitalization and mortality [3]. Flu-p can progress from relatively mild disease to more severe cases that can cause ARDS or respiratory failure such that patients must often undergo invasive mechanical ventilation (IMV) within 3–10 days following initial symptom onset [4, 5]. The need for IMV is linked to higher rates of patient morbidity and mortality [6], and evaluating a given patient’s odds of requiring IMV is thus a valuable prognostic approach. However, the risk factors associated with the need for IMV have not been fully clarified. Certain assessment tools have been established in an effort to gauge the odds of IMV in individuals suffering from acute hypoxemic respiratory failure, including the ROX index (pulse oximetry/FiO_2_ to respiratory rate) [7], modified ROX index (PaO_2_/FiO_2_ to respiratory rate) [8], and HACOR scale (heart rate, respiratory rate, arterial pH, PaO_2_/FiO_2_ and Glasgow Coma Scale) [9]. These tools, however, are not specific to Flu-p patients, nor have any studies specifically examined their predictive power in individuals suffering from Flu-p, and there is thus a clear need for the development of a reliable tool that can predict the requirement for IMV in Flu-p patients at an early time point prior to the onset of potential respiratory failure.

As such, we performed the present retrospective multicenter study with the goal of developing an accurate and easy-to-use assessment tool capable of predicting the odds of a given Flu-p patient undergoing IMV within 14 days of admission.

## Methods

### Patient recruitment

For this study, patients for whom influenza virus nucleic acid testing was performed in the microbiology labs of five tertiary hospitals in China (Additional file 1: Supplementary Material 1) from January 1st, 2012 to December 31st, 2019 were screened for eligibility. Those patients with confirmed Flu-p were enrolled in this study. Patients were excluded if they: (1) were not classified as having community-onset pneumonia (with pneumonia onset ≥ 48 h after admission [10]), as the inclusion of nosocomial pneumonia cases had the potential to complicate result interpretations; (2) were < 18 years old; (3) were immunocompromised, given that the clinical features and outcomes of immunocompromised patients were different from those of immunocompetent hosts [11]; or (4 had been intubated prior to admission.

### Study definitions

Patients with Flu-p were defined as individuals for whom polymerase chain reaction (PCR) analyses of respiratory specimens (including sputum, nasal/nasopharyngeal swabs, bronchial aspirates, and bronchoalveolar lavage fluid) were positive for influenza viral RNA, and for whom respiratory symptoms and chest radiographic findings were consistent with newly emergent chest infiltrates. The decisions to initiate IMV were taken by the attending physicians, based on the presence of any of the following intubation criteria: respiratory or cardiac arrest, respiratory pauses with loss of alertness or gasping for air, severely impaired consciousness, major agitation inadequately controlled by sedation, signs of exhaustion, massive aspiration, inability to manage respiratory secretions appropriately, and hemodynamic instability without response to fluids and vasoactive agents. Additionally, patients were also intubated in case of subsequent worsening of gas exchange or respiratory distress despite supportive measures [12]. Early neuraminidase inhibitor (NAI) therapy was defined as the administration of NAI agents within two days of symptom onset [13]. Systemic corticosteroid treatment was defined by the administration of one or more systemic corticosteroid doses at admission. Community-acquired co-infecting respiratory pathogens were defined as pathogens detected via standard microbiological techniques (Additional file 1: Supplementary Material 2) within 48 h following admission [14].

### Data collection

Data extracted from patient medical records with a standard case report form included demographic details, patient comorbidities (see Additional file 1: Supplementary Material 3), patient symptoms, vital signs, laboratory results, radiographic findings before invasive ventilation at the day of admission (if there were multiple results, the worst value was extracted), community-acquired co-infecting respiratory pathogens, patient management, and outcome data (including NAI use, systemic corticosteroid administration, IMV, and 14-day mortality). Outcomes for those hospitalized for < 14 days were established through follow-up via telephone.

### Statistical analysis

In total, 1107 Flu-p patients were identified and enrolled in this study. These patients were then randomly assigned to derivation and validation cohorts (80% and 20% of patients, respectively), which were respectively used to develop and validate our prognostic model.

In addition, the patients in the derivation cohort were separated into two groups based upon whether or not they underwent IMV within 14 days following admission. Baseline characteristics were then compared between these two patient groups, and all variables which yielded a *p* < 0.1 in these initial univariable analyses were incorporated into a multivariable backward stepwise logistic regression model to identify risk factors associated with 14-day IMV rates. To ensure model simplicity, each risk factor was assigned an integer score value associated with its corresponding regression coefficient (β) value. Model cutoff scores were then defined using receiver operating characteristic (ROC) curves based upon Youden’s index. Kaplan–Meier analyses were conducted to compare rates of IMV between patients above and below this cutoff score (high-risk and low-risk, respectively). The area under the ROC curve (AUROC) was used to gauge the prognostic performance of this model based upon overall sensitivity and specificity values. Model calibration was assessed by comparing the predicted risk of IMV with the observed risks, and examined using the Hosmer–Lemeshow goodness-of-fit statistic.

To handle the missing data, multiple imputation was first performed by chained equations, which use linear regression to impute continuous variables and (multinomial) logistic regression to impute categorical/binary variables [15]. The interaction of independent variables was checked by a multicollinearity test using the variance inflation factor (VIF), and no problem was detected (0 < VIF < 10).

A Kolmogorov–Smirnov test was used to assess result normality, with normally and non-normally distributed variables being presented as means ± standard deviation and medians, respectively. Continuous variables were evaluated with Mann–Whitney *U* tests or Student’s t-tests, whereas categorical variables were assessed with Fisher’s exact test or chi-squared tests. A two-tailed *p* < 0.05 was indicative of significance. SPSS 22.0 or MedCalc 19.0 were used for all statistical testing.

## Results

### Patient screening

**Fig. 1:**
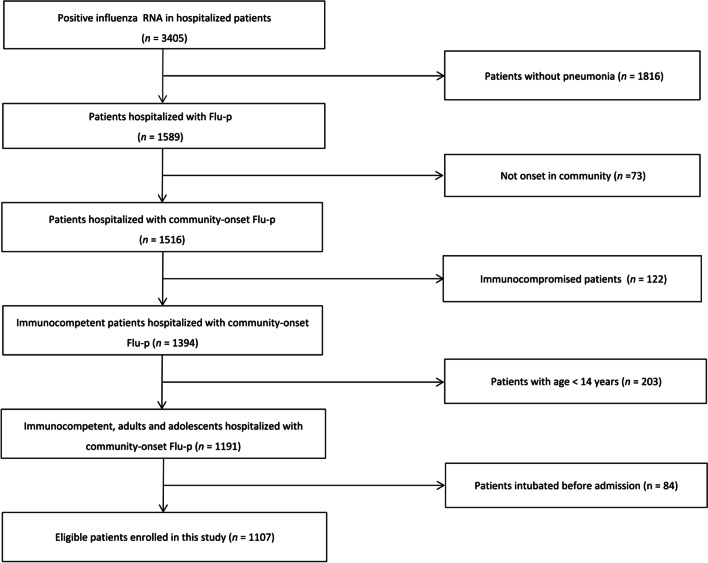
Patient screening algorithm for Flu-p. A total of 3405 patients were screened, and 1107 patients with laboratory-confirmed Flu-p were enrolled in this study

A total of 3405 hospitalized patients who were found to be positive for influenza viral RNA during the study period were screened for eligibility, of whom 1107 with laboratory-confirmed Flu-p were enrolled in this study (Fig. 1).

### Patient characteristics

Enrolled patients exhibited a median age of 61.0 years, and were 54.5% (603/1107) male. The most prevalent comorbidities in these patients included cardiovascular disease (22.7%, 251/1107), diabetes mellitus (13.4%, 148/1107), and cerebrovascular disease (10.3%, 114/1107) (Additional file 1: Supplementary material 4). In total, 33.5% (371/1107) patients were found to be co-infected with other community-acquired pathogens (Additional file 1: Supplementary Material 5). Of these patients, non-invasive mechanical ventilation (NIMV) and IMV were respectively conducted within 14 days of admission for 11.6% (128/1107) and 10.6% (117/1107) of patients. The all-cause 14-day mortality rate for these patients was 3.2% (35/1107) (Additional file 1: Supplementary material 4).

### Risk factors associated with 14-day IMV rates in Flu-p patients

Next, univariable analyses were conducted in the derivation cohort, which identified age ≥ 65 years old, influenza A virus infection, the presence of solid malignant tumors, a respiratory rate ≥ 30 breaths/min, a leukocyte count > 10 × 10^9^/L, Lymphocytes < 0.8 × 10^9^/L, albumin < 35 g/L, arterial PH < 7.35, PaO_2_/FiO_2_ < 300 mmHg, early NAI therapy, and systemic corticosteroids use at admission associated with 14-day IMV rates in Flu-p patients (Table 1).

**Table 1:** Comparison of clinical characteristics and outcomes between patients needing and weaning from IMV from derivation cohort

Variable	Needing IMV (*n* = 99)	Weaning from IMV (*n* = 796)	*p* Value
Age ≥ 65 years old (*n*, %) ^#^	66 (66.7)	334 (42.0)	** < 0.001**
Male (*n*, %)	55 (55.6)	421 (52.9)	0.616
Influenza A infection (*n*, %) ^#^	72 (72.7)	471 (59.2)	**0.009**
Comorbidities (*n*, %)			
Cardiovascular disease	26 (26.3)	176 (22.1)	0.351
Diabetes mellitus	11 (11.1)	102 (12.8)	0.630
Cerebrovascular disease	9 (9.1)	78 (9.8)	0.823
COPD	10 (10.1)	75 (9.4)	0.828
Chronic kidney disease	0 (0.0)	29 (3.6)	0.103
Asthma	1 (1.0)	23 (2.9)	0.446
Solid Malignant tumor ^#^	8 (8.1)	13 (1.6)	** < 0.001**
Pregnancy (*n*, %)	0 (0.0)	6 (0.8)	1.000
Obesity (*n*, %)	4 (4.0)	53 (6.7)	0.314
Smoking history (*n*, %)	24 (24.2)	215 (27.0)	0.557
Baseline clinical and radiologic features (*n*, %)			
Mental confusion	15 (15.2)	96 (12.1)	0.379
Respiratory rates ≥ 30 breaths/min ^#^	54 (54.5)	77 (9.7)	** < 0.001**
SBP < 90 mmHg	1 (1.0)	6 (0.8)	0.561
Leukocytes > 10 × 10^9^/L ^#^	14 (14.1)	203 (25.5)	**0.013**
Lymphocytes < 0.8 × 10^9^/L ^#^	67 (67.7)	312 (39.2)	** < 0.001**
HB < 100 g/L	18 (18.2)	179 (22.5)	0.329
ALB < 35 g/L ^#^	10 (10.1)	153 (19.2)	**0.014**
BUN > 7 mmol/L	35 (35.4)	316 (39.7)	0.386
BG > 14 mmol/L	0 (0.0)	8 (1.0)	0.606
Arterial PH < 7.35 ^#^	21 (22.2)	67 (8.4)	**0.001**
PaO_2_/FiO_2_ < 300 mmHg ^#^	81 (81.8)	351 (44.1)	** < 0.001**
PaCO_2_ > 50 mmHg	7 (7.1)	82 (10.3)	0.133
Multilobar infiltrates ^#^	77 (77.8)	558 (70.1)	0.113
Pleural effusion	27 (27.3)	251 (31.5)	0.388
Coinfections (*n*, %)	32 (32.3)	270 (33.9)	0.751
Early NAI therapy (*n*, %) ^#^	17 (17.2)	294 (36.9)	** < 0.001**
Systemic corticosteroids use at admission (*n*, %) ^#^	21 (21.2)	32 (4.0)	** < 0.001**
Noninvasive ventilation within 14 days after admission (*n*, %)	17 (17.2)	94 (11.8)	0.127

*IMV* invasive mechanical ventilation, *NIMV* noninvasive mechanical ventilation, *COPD* chronic obstructive pulmonary disease, *SBP* systolic blood pressure, *HB* hemoglobin, *ALB* albumin, *BUN* blood urea nitrogen, *BG* blood glucose, *PaO*_*2*_*/FiO*_*2*_ arterial pressure of oxygen/fraction of inspiration oxygen, *NAI* neuraminidase inhibitor. #: variables cited in the table above were the candidates which were entered into the multivariate logistic regression model. The bolded values are *p* values < 0.05, which represented significant differences between patients needing and free of invasive ventilation

**Fig. 2:**
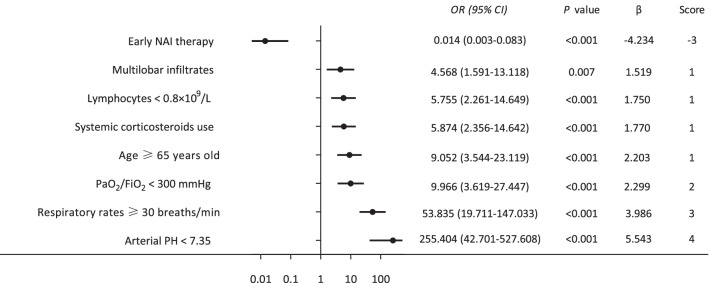
Risk factors associated with invasive mechanical ventilation in Flu-p patients. A multivariate logistic regression model identified the following factors as independent predictors of a higher risk of requiring IMV in Flu-p patients: early NAI use, lymphocytes < 0.8 × 10^9^/L, multilobar infiltrates, age ≥ 65 years old, systemic corticosteroid administration, PaO_2_/FiO_2_ < 300 mmHg, respiratory rate ≥ 30 breaths/min, and arterial PH < 7.35

In a subsequent multivariable logistic regression model, factors that were identified as independent predictors of a higher risk of requiring IMV in Flu-p patients (Fig. 2) included: early NAI use (*OR* 0.014, 95% *CI* 0.003–0.083, *p* < 0.001; −3 points), multilobar infiltrates (*OR* 4.568, 95% *CI* 1.591–13.118, *p* = 0.007; 1 point), lymphocytes < 0.8 × 10^9^/L (*OR* 5.755, 95% CI 2.261–14.649, *p* < 0.001; 1 point), systemic corticosteroid administration (*OR* 5.874, *95% CI* 2.356–14.642, *p* < 0.001; 1 points), age ≥ 65 years old (*OR* 9.052, 95% *CI* 3.544–23.119, *p* < 0.001; 1 points), PaO_2_/FiO_2_ < 300 mmHg (*OR* 9.966, 95% *CI* 3.619–27.447, *p* < 0.001; 2 points), respiratory rate ≥ 30 breaths/min (*OR* 53.835, 95% *CI* 19.711–147.033, *p* < 0.001; 3 points), and arterial PH < 7.35 (*OR* 255.404, 95% *CI* 42.701–527.608, *p* < 0.001; 4 points).

### Assessment of the predictive performance of Flu-IV scores

**Fig. 3:**
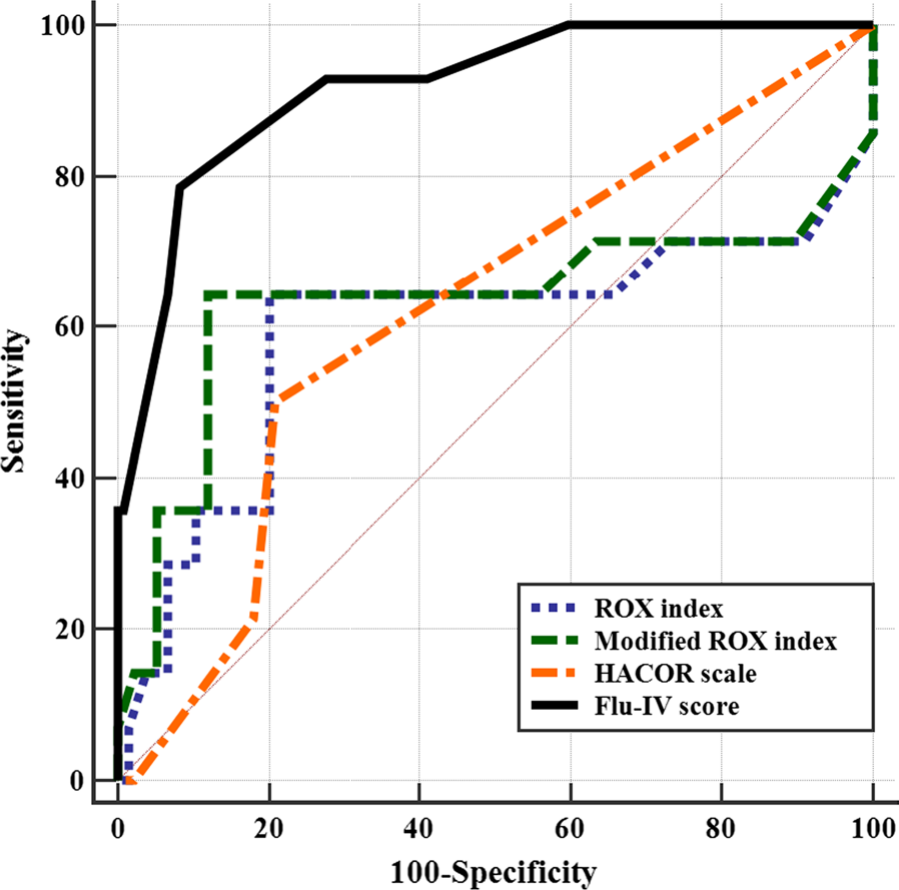
ROCs for IMV prediction of four scorings in Flu-p patients from validation cohort. The AUROC value for the Flu-IV score model was 0.909, which was higher than that of the ROX index (AUROC = 0.594), modified ROX index (AUROC = 0.633) and HACOR scale (AUROC = 0.622)

The AUROC value for the Flu-IV score model developed based upon the above multivariable analysis was 0.909 in our validation patient cohort (95% *CI* 0.851–0.950), and was higher than that of the ROX index (AUROC = 0.594, 95% CI 0.510–0.674, *p* = 0.004), modified ROX index (AUROC = 0.633, 95% *CI* 0.550–0.710, *p* = 0.012), or HACOR scale (AUROC = 0.622, 95% *CI* 0.539–0.700, *p* < 0.001) (Additional file 1: Supplementary Material 6 and Fig. 3). Similar findings were also independently made in the derivation cohort (Additional file 1: Supplementary Material 7 and Supplementary Fig. 1).

A good performance was observed between the observed and predicted risk of IMV from the derivation cohort and validation cohort, indicating that the algorithms were well calibrated (Additional file 1: Supplementary Fig. 2).

**Fig. 4:**
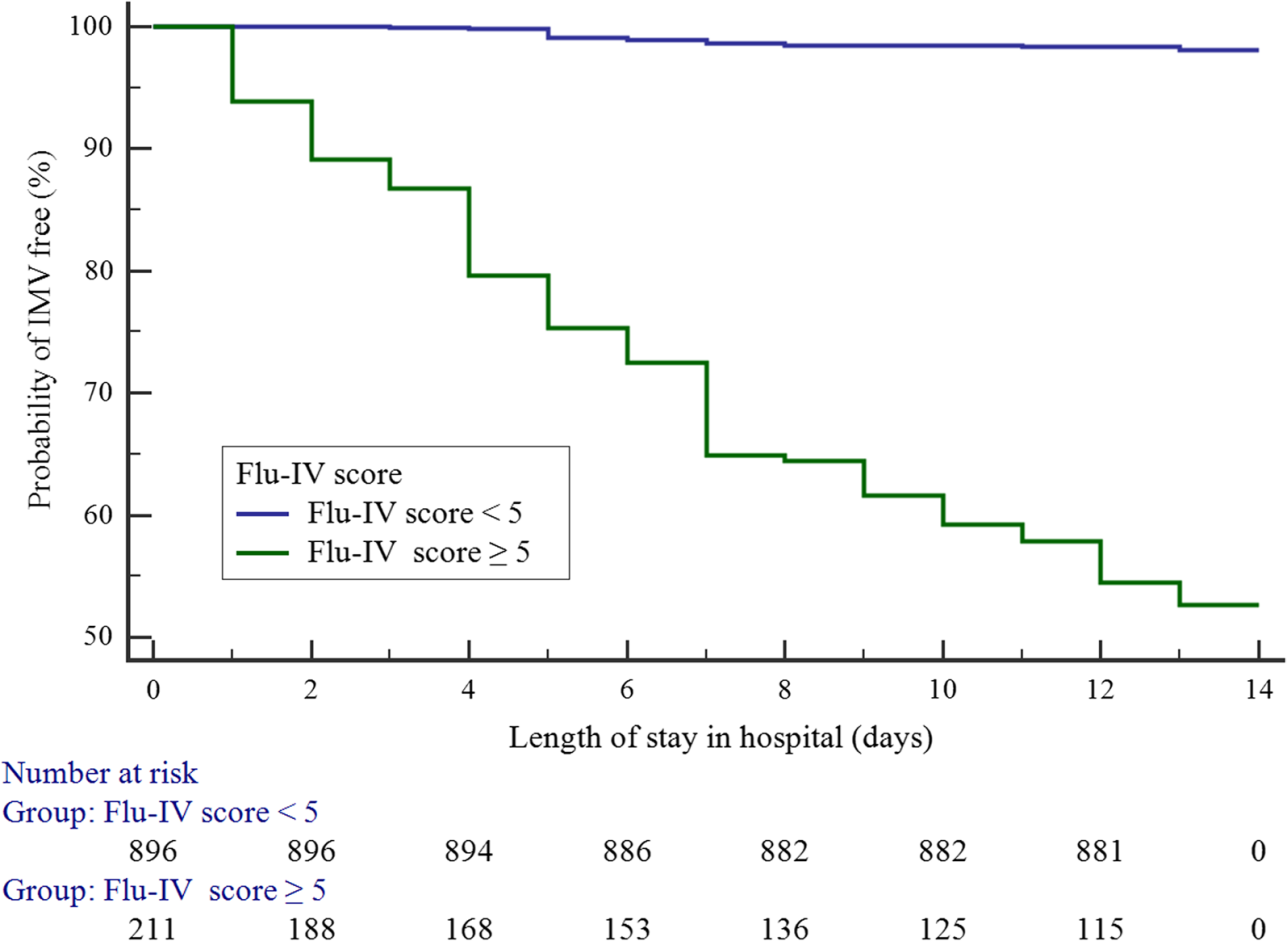
Kaplan–Meier curve showing the probability of IMV according to the Flu-IV score (Flu-IV score < 5: Low risk; Flu-IV score ≥ 5: High risk). Patients with Flu-Iv score ≥ 5 were significantly more likely to require IMV relative to low-risk patients (47.4% vs. 1.9%, log-rank test, *p* < 0.001)

Table 2 compiles the mortality rates, sensitivity, and specificity values associated with our Flu-IV score model in the overall patient cohort. Patients were stratified into high- and low-risk cohorts based upon whether they had Flu-IV scores that were above or below the optimal cutoff score of 5 points. Subsequent Kaplan–Meier curves confirmed that high-risk patients were significantly more likely to require IMV relative to low-risk patients (47.4% vs. 1.9%, log-rank test, *p* < 0.001) (Fig. 4).

**Table 2:** Flu-IV score and actual proportion of patients needing IMV

Score	Cases of IMV (*n*, %)	Sensitivity (%)	95% *CI*	Specificity (%)	95% *CI*	+LR	−LR
− 3	0/11 (0.0)	100.00	96.9–100.0	0.00	0.0–0.5	1.00	
− 2	0/58 (0.0)	100.00	96.9–100.0	1.11	0.6–2.0	1.01	0.00
− 1	1/64 (1.6)	100.00	96.9–100.0	6.97	5.5–8.7	1.07	0.00
0	1/98 (1.0)	99.15	95.3–100.0	13.33	11.3–15.6	1.14	0.064
1	1/149 (0.7)	98.29	94.0–99.8	23.13	20.5–25.9	1.28	0.074
2	1/124 (0.8)	97.44	92.7–99.5	38.08	35.0–41.2	1.57	0.067
3	9/235 (3.8)	96.58	91.5–99.1	50.51	47.3–53.7	1.95	0.068
4	4/157 (2.5)	88.89	81.7–93.9	73.33	70.5–76.1	3.33	0.15
**5**	27/82 (32.9)	85.47	77.8–91.3	88.79	86.7–90.7	7.62	0.16
6	23/71 (32.4)	62.39	53.0–71.2	94.34	92.7–95.7	11.03	0.40
7	15/23 (65.2)	42.74	33.6–52.2	99.19	98.4–99.7	52.88	0.58
8	35/35 (100.0)	29.91	21.8–39.1	100.00	99.6–100.0		0.70
9	–	0.00	0.0–3.1	100.00	99.6–100.0		1.00

*CI* confidence interval, +*LR* positive likelihood ratio, −*LR* negative likelihood ratio

## Discussion

This was a multicenter retrospective study designed to develop a novel model capable of predicting the odds of IMV within 14 days of admission for Flu-p patients. Our resultant Flu-IV risk score model can be used to reliably predict 14-day IMV rates in Flu-p patients.

We found that 10.6% of the patients in the present study necessitated IMV within 14 days of admission, in line with prior reports regarding severe influenza patient outcomes [16, 17]. The 14-day mortality of patients that did require IMV was significantly greater than that of patients that did not. As 92.1% of Flu-p patients that undergo IMV do so within 14 days of admission, predicting 14-day IMV rates is critical to appropriate patient management.

We identified multiple variables that are known to be associated with more severe influenza and that were also associated with a higher risk of IMV in Flu-p patients [18], including age > 65 years, a lymphocyte count < 0.8 × 10^9^/L, and systemic corticosteroid use. Cellular immunity is a key mediator of antiviral responses [19], and advanced age is associated with a decline in overall patient immune status [20]. Severe influenza is also often characterized by lymphocytopenia in 50–100% of cases [21, 22], although the mechanistic basis for this finding remains poorly understood. There is some evidence that CD4+ and CD8+ T cells may undergo higher rates of apoptotic death in individuals with severe disease owing to higher circulating levels of soluble Fas ligand and caspase-1 [23], thereby contributing to an overall decline in lymphocyte counts. Such virus-induced lymphocytopenia can delay viral clearance. Alternatively, these lymphocytes may be recruited to the respiratory tract and other organs, resulting in their apparent depletion from circulation [24]. Lymphocyte accumulation within the lungs can drive more severe localized inflammation and tissue damage. Systemic corticosteroid use can suppress overall immune functionality and increase the odds of developing severe nosocomial pneumonia necessitating IMV [25, 26].

Severe Flu-p is characterized by impaired pulmonary function and diffuse alveolar damage [27], with tachypnea and decreased PaO_2_/FiO_2_ serving as direct manifestations of such pulmonary damage. Pneumonia patients also often exhibit metabolic acidosis that is linked to hyper-inflammation and impaired tissue perfusion [28], thereby exacerbating pulmonary damage. Impaired pulmonary function and the retention of carbon dioxide in the lungs can further drive respiratory acidosis, leading to higher rates of NIMV failure and an increased risk of requiring IMV [29]. Many observational studies showed inhibiting viral replication at early time points can reduce virus-induced inflammation and tissue damage, thereby decreasing overall influenza-related mortality rates [30–32]. Our data also suggest that early NAI treatment was associated with a lower risk of Flu-p patient intubation.

The ROX/modified ROX index are designed to predict failure of high flow oxygen by nasal cannula at 2, 6 and 12 h after start. The HACOR scale predicts non-invasive ventilation (NIV) failure within 1 h after the start of NIV. The time window and intended use of these scores differ to our Flu-IV substantially. In addition, the ROX/modified ROX and the HACOR scale are only applied to the patients suffering from hypoxemia. However, just 49% of patients in the present study cohort exhibited hypoxemia upon admission. Importantly, these scoring systems were not designed for the analysis of Flu-p patients. While some of the variables included in our Flu-IV model were the same as those included in the ROX, modified ROX, and HACOR scales, these tools were not able to reliably predict IMV rates among Flu-p patients at the day of admission. A Flu-IV cutoff score of 5 was able to effectively stratify Flu-p patients into low- and high-risk categories. Considering its good negative prediction value, the Flu-IV score could be used particularly as a rule-out approach to early discharge patient with a low score. Importantly, our Flu-IV scoring model is simple, allowing clinicians to predict the odds of a given patient requiring IMV within 14 days of admission based upon eight parameters that can be readily measured even in small or primary hospitals. This model can be used to evaluate patients at an early time point prior to the onset of respiratory failure, and as such, we believe it represents a valuable tool for the management of Flu-p patients.

There are certain limitations to this analysis. For one, as this study was retrospective in design, it is susceptible to selection bias. Nucleic acid tests, for example, we conducted based upon the subjective judgment of the attending physicians such that only patients with more severe disease may have undergone such testing, rather than all potentially eligible patients. Furthermore, it was possible that a very few patients required IMV did not consent to intubation. As this study was retrospective, we were also unable to retrieve and evaluate patient vaccination data or other missing information, potentially constraining the accuracy of our findings. Patients were also not routinely evaluated for other respiratory viruses, and we are thus unable to exclude the possibility that certain patients may have been co-infected with multiple viruses.

## Conclusions

In summary, we developed a reliable and straightforward predictive tool capable of gauging the odds of a given hospitalized Flu-p patient requiring IMV. This tool will help clinicians better evaluate the risks of early intubation for any given patient such that they can make optimal clinical judgments. However, it should be evaluated in more large-sample and prospective studies.

## Supplementary Information

**Additional file 1.** Detailed and additional data of this manuscript.

## Data Availability

All data generated or analysed during this study are included in this published article and its Additional file 1.

## Abbreviations

IMV: Invasive mechanical ventilation
Flu-p: Influenza-related pneumonia
ROX index: Pulse oximetry/FiO_2_ to respiratory rate
modified ROX index: PaO_2_/FiO_2_ to respiratory rate
HACOR scale: Heart rate, respiratory rate, arterial pH, PaO_2_/FiO_2_ and Glasgow Coma Scale
PCR: Polymerase chain reaction
NAI: Neuraminidase inhibitor
OR: Odds ratio
95% IC: 95% Interval confidence
IQR: Interquartile range
ROC: Receiver operating characteristic
AUROC: Area under the ROC curve
COPD: Chronic obstructive pulmonary disease
SBP: Systolic blood pressure
Hb: Hemoglobin
ALB: Albumin
BUN: Blood urea nitrogen
PH: Hydrogen ion index
pO_2_/FiO_2_: Arterial pressure of oxygen/fraction of inspiration oxygen
