# Supplementary material for: Flu-IV score: a predictive tool for assessing the risk of invasive mechanical ventilation in patients with influenza-related pneumonia
Source: BMC Pulm Med. 2022 Jan 29;22:47. doi: 10.1186/s12890-022-01833-2 (PMC8799963; doi:10.1186/s12890-022-01833-2)
Supplement: Supplementary file 1 — Additional file 1. Detailed and additional data of this manuscript. [file 12890_2022_1833_MOESM1_ESM.docx]

**Supplementary Material 1: Details of participating centers**

| **Name of the hospital** | **Province, city** | **Teaching Hospital** | **Beds** | **Staffs of Clinical Microbioloy Lab** |
| --- | --- | --- | --- | --- |
| Beijing Jishuitan Hospital | Beijing | Yes | 1500 | 10 |
| Beijing Chao-Yang Hospital | Beijing | Yes | 1400 | 11 |
| **the 2nd People’s Hospital of Yunnan Province** | **Kunming,**  **Yan’an** | Yes | 1302 | 4 |
| **Qingdao Municipal Hospital** | ShanDong,  Qingdao | Yes | 1200 | 4 |
| Beijing Huimin Hospital | Beijing | Yes | 1000 | 2 |

**Supplementary Material 2 Definition of microbiological criteria of coinfected with other pathogens**

1. Positive urinary antigen for *Legionella pneumophila*;
2. Positive urinary antigen for *Streptococcus pneumoniae*;
3. Positive bacterial culture from blood or plural fluid except for coagulase negative *Staphylococcus spp*.;
4. Paired sera with a fourfold or more increase in the titers of antibodies to *Mycoplasma pneumoniae* (MP), *Chlamydia pneumonia*, *L pneumophila or* respiratory viruses ( Parainfluenza, Adenovirus, Respiratory syncytial virus)*.* Or Serum IgM antibody (MIF) ≥ 1:16 for *Chlamydia pneumonia*;
5. Detection of respiratory virus in sputum/bronchoalveolar lavage (BALF)/throat swabs by Realtime-PCR according to manufacturer’s instructions, including respiratory syncytial virus (RSV) types A and B, parainfluenza virus (PIV) types 1, 2, 3 and 4, rhinovirus (HRV), enterovirus (EV), coronavirus (hCoV) types 229E, NL63, OC43 and HKU1, parapneumovirus (hMPV), and adenovirus (AdV), bocavirus;
6. Bacteria isolated form purulent sputum (defined as an adequate quality sputum sample with > 25 leukocytes and < 10 epithelial cells per × 100 magnification field) with compatible findings of Gram staining;
7. Detection of *Mycoplasma pneumoniae* (MP), *Chlamydia pneumonia* or *L pneumophila* in sputum/BALF/throat swabs by Realtime-PCR;
8. serum IgM antibody positive for *Mycoplasma pneumoniae* (MP), or Serum IgG antibody (MIF) ≥ 1:512 for *Chlamydia pneumonia;*
9. Invasive pulmonary aspergillosis were diagnosed in accordance with the revised definitions of invasive fungal diseases from the European Organization for Research and Treatment of Cancer and the Mycoses Study Group Education and Research Consortium [1].

References

1.Donnelly JP, Chen SC, Kauffman CA, et al. Revision and Update of the Consensus Definitions of Invasive Fungal Disease From the European Organization for Research and Treatment of Cancer and the Mycoses Study Group Education and Research Consortium. Clin Infect Dis. 2019, pii: ciz1008.

**Supplementary Material 3 Definition of underlying diseases**

1. Smoking was defined as cigarette smokers of 10 cigarettes/d during at least the previous year；
2. Hypertension was defined as systolic blood pressure≥140mmHg and /or diastolic blood pressure ≥ 90 mmHg in resting status;
3. Chronic pulmonary disease was defined as: persistent airflow limitation, FEV_1_ / FVC < 70% post bronchodilator;
4. Asthma was defined by the history of respiratory symptoms such as wheeze, cough that varied over time and intensity, together with variable respiratory airway limitation;
5. Cardiovascular disease included coronary heart disease and chronic congestive heart failure;
6. Coronary heart disease included angina pectoris, myocardial infarction, ischemic cardiomyopathy;
7. Chronic congestive heart failure was defined as cardiomegaly and ejection fraction ≤ 40%;
8. Cerebrovascular diseases included transient ischemic attack, cerebral hemorrhage, subarachnoid hemorrhage, cerebral infarction;
9. Diabetes mellitus: included diabetes mellitus type 1 and diabetes mellitus type 2, not included impaired glucose tolerance and impaired fasting glycaemia;
10. Chronic kidney disease included diabetic nephropathy, hypertensive renal damage, chronic glomerulonephritis, chronic pyelonephritis, lupus nephritis, IgA nephropathy, nephrotic syndrome, hereditary kidney disease;
11. Obesity was defined as body mass index (BMI) ≥ 30 kg/m^2^;
12. Immunosuppressive therapy: was defined as systmetic glucocorticosteroid (such as prednisone ≥ 10mg/d for more than 3 weeks in the last month); cyclosporine or azathioprine use within 3 months, and methotrexate use ≥ 12.5 mg/week within 3 months; biological modifiers such as etanercept and infiximab within 3 weeks.
13. Immunocompromised status included primary immune deficiency diseases, active malignancy, HIV infection with a CD4 T-lymphocyte count < 200 cells/mL or percentage < 14%, immunosuppressive therapy, solid organ transplantation, hematopoietic stem cell transplantation, splenectomy [1].
14. Mental confusion was defined as a mental test score of 8 or less or disorientation in person, place or time.
15. Ramirez JA, Musher DM, Evans SE, Dela Cruz C, Crothers KA, Hage CA, Aliberti S, Anzueto A, Arancibia F, Arnold F et al: Treatment of Community-Acquired Pneumonia in Immunocompromised Adults: A Consensus Statement Regarding Initial Strategies. Chest 2020, 158(5):1896-1911

**Supplementary Material 4 Comparison of baseline clinical characteristics and outcomes between patients in the derivation and validation cohorts**

| **Variable** | **Total**  **(*n* = 1107)** | **Derivation Cohort**  **(*n* = 895)** | **Validation Cohort**  **(*n* = 212)** |
| --- | --- | --- | --- |
| Age (years, median, IQR) | 61.0 (52.0-77.0) | 61.0 (52.0-77.0) | 61.0 (49.0-78.8) |
| ≥ 65 years old (*n*, %) | 497 (44.9) | 400 (44.7) | 97 (45.8) |
| Male (*n*, %) | 603 (54.5) | 476 (53.2) | 127 (59.9) |
| Influenza A infection (*n*, %) | 683 (61.7) | 543 (60.7) | 140 (66.0) |
| Chronic medical conditions (*n*, %) |  |  |  |
| Cardiovascular disease | 251 (22.7) | 202 (22.6) | 49 (23.1) |
| Diabetes mellitus | 148 (13.4) | 113 (12.6) | 35 (16.5) |
| Cerebrovascular disease | 114 (10.3) | 87 (9.7) | 27 (12.7) |
| COPD | 102 (9.2) | 85 (9.5) | 17 (8.0) |
| Chronic kidney disease | 35 (3.2) | 29 (3.2) | 6 (2.8) |
| Asthma | 27 (2.4) | 24 (2.7) | 3 (1.4) |
| Solid Malignant tumor | 27 (2.4) | 21 (2.3) | 6 (2.8) |
| Obesity (*n*, %) | 73 (6.6) | 57 (6.4) | 16 (7.5) |
| Pregnancy (*n*, %) | 9 (0.8) | 6 (0.7) | 3 (1.4) |
| Smoking history (*n*, %) | 302 (27.3) | 239 (26.7) | 63 (29.7) |
| Baseline clinical and radiological features (*n*, %) |  |  |  |
| Mental confusion | 129 (11.7) | 111 (12.4) | 18 (8.5) |
| Respiratory rates ≥ 30 breaths/min | 159 (14.4) | 131 (14.6) | 28 (13.2) |
| SBP < 90 mmHg | 9 (0.8) | 7 (0.8) | 2 (0.9) |
| Leukocytes > 10×10^9^/L | 270 (24.4) | 217 (24.2) | 53 (25.0) |
| Lymphocytes < 0.8×10^9^/L | 465 (42.0) | 379 (42.3) | 86 (40.6) |
| HB < 100 g/L | 237 (21.4) | 197 (22.0) | 40 (18.9) |
| ALB < 35 g/L | 202 (18.2) | 163 (18.2) | 39 (18.4) |
| BUN > 7 mmol/L | 414 (37.4) | 351 (39.2) | 63 (29.7) |
| BG > 14 mmol/L | 10/1034 (1.0) | 8/837 (1.0) | 2/197 (1.0) |
| Arterial PH < 7.35 | 103 (9.3) | 88 (9.8) | 15 (7.1) |
| PaO_2_/FiO_2_ < 300 mmHg | 537 (48.5) | 432 (48.3) | 105 (49.5) |
| Multilobar infiltrates (*n*, %) | 789 (71.3) | 635 (70.9) | 154 (72.6) |
| Pleural effusion (*n*, %) | 343 (31.0) | 278 (31.1) | 65 (30.7) |
| Coinfections (*n*, %) | 371 (33.5) | 302 (33.7) | 69 (32.5) |
| Early NAI therapy (*n*, %) | 391 (35.3) | 311 (34.7) | 80 (37.7) |
| Systemic corticosteroid use at admission (*n*, %) | 68 (6.1) | 53 (5.9) | 15 (7.1) |
| Noninvasive ventilation within 14 days after admission (*n*, %) | 128 (11.6) | 111 (12.4) | 17 (8.0) |
| Invasive ventilation within 14 days after admission (*n*, %) | 117 (10.6) | 99 (11.1) | 18 (8.5) |
| 14-day mortality (*n*, %) | 35 (3.2) | 26 (2.9) | 9 (4.2) |

IQR: interquartile range; SD: standard deviation; COPD: chronic obstructive pulmonary disease; SBP: systolic blood pressure; HB: hemoglobin; ALB: albumin; BUN: blood urea nitrogen; BG: blood glucose; pO_2_/FiO_2_: arterial pressure of oxygen/fraction of inspiration oxygen; NAI: neuraminidase inhibitor.

**Supplementary Material 5 coinfections with other community-acquired pathogens**

| **Variable** | **Total**  **(*n* = 1107)** |
| --- | --- |
| Coinfection (*n*, %) | 371 (33.5) |
| Pathogens (*n*, %) |  |
| *Klebsiella pneumoniae* | 121 (32.6) |
| *Streptococcus pneumoniae* | 111 (29.9) |
| *Staphylococcus aureus* | 73 (19.7) |
| *Haemophilus influenzae* | 30 (8.1) |
| *Other streptococcus spp.* | 16 (4.3) |
| *Klebsiella acidogens* | 9 (2.4) |
| *Escherichia coli* | 5 (1.3) |
| *Pseudomonas aeruginosa* | 4 (1.1) |
| *Proteus spp.* | 4 (1.1) |
| *Citrobacter spp.* | 3 (0.8) |
| *Flavobacterium* | 2 (0.5) |
| *Aspergillus spp.* | 2 (0.5) |
| *Stenotrophomonas maltophilia* | 1 (0.3) |
| *Acinetobacter* | 1 (0.3) |

11 patients were coinfected with 2 or more pathogens

**Supplementary Material 6 AUC for IMV prediction in Flu-p patients from validation cohort**

| **Variable** | **AUC** | **SE** | ***95% CI*** | ***Z* statistic** | ***p* value** |
| --- | --- | --- | --- | --- | --- |
| Flu-IV score | 0.909 | 0.040 | 0.851 to 0.950 | —— | Reference |
| ROX index | 0.594 | 0.111 | 0.510 to 0.674 | 2.900 | 0.004 |
| Modified ROX index | 0.633 | 0.114 | 0.550 to 0.710 | 2.524 | 0.012 |
| HACOR scale | 0.622 | 0.067 | 0.539 to 0.700 | 4.111 | < 0.001 |

AUC: area under the curve; SE: standard error; CI:confidence interval.

**Supplementary Material 7 AUC for IMV prediction in Flu-p patients from derivation cohort**

| **Variable** | **AUC** | **SE** | ***95% CI*** | ***Z* statistic** | ***p* value** |
| --- | --- | --- | --- | --- | --- |
| Flu-IV score | 0.912 | 0.018 | 0.887 to 0.933 | —— | Reference |
| ROX index | 0.679 | 0.038 | 0.641 to 0.715 | 6.434 | < 0.001 |
| Modified ROX index | 0.745 | 0.039 | 0.709 to 0.778 | 4.523 | < 0.001 |
| HACOR scale | 0.509 | 0.029 | 0.470 to 0.549 | 11.231 | < 0.001 |

AUC: area under the curve; SE: standard error; CI:confidence interval.


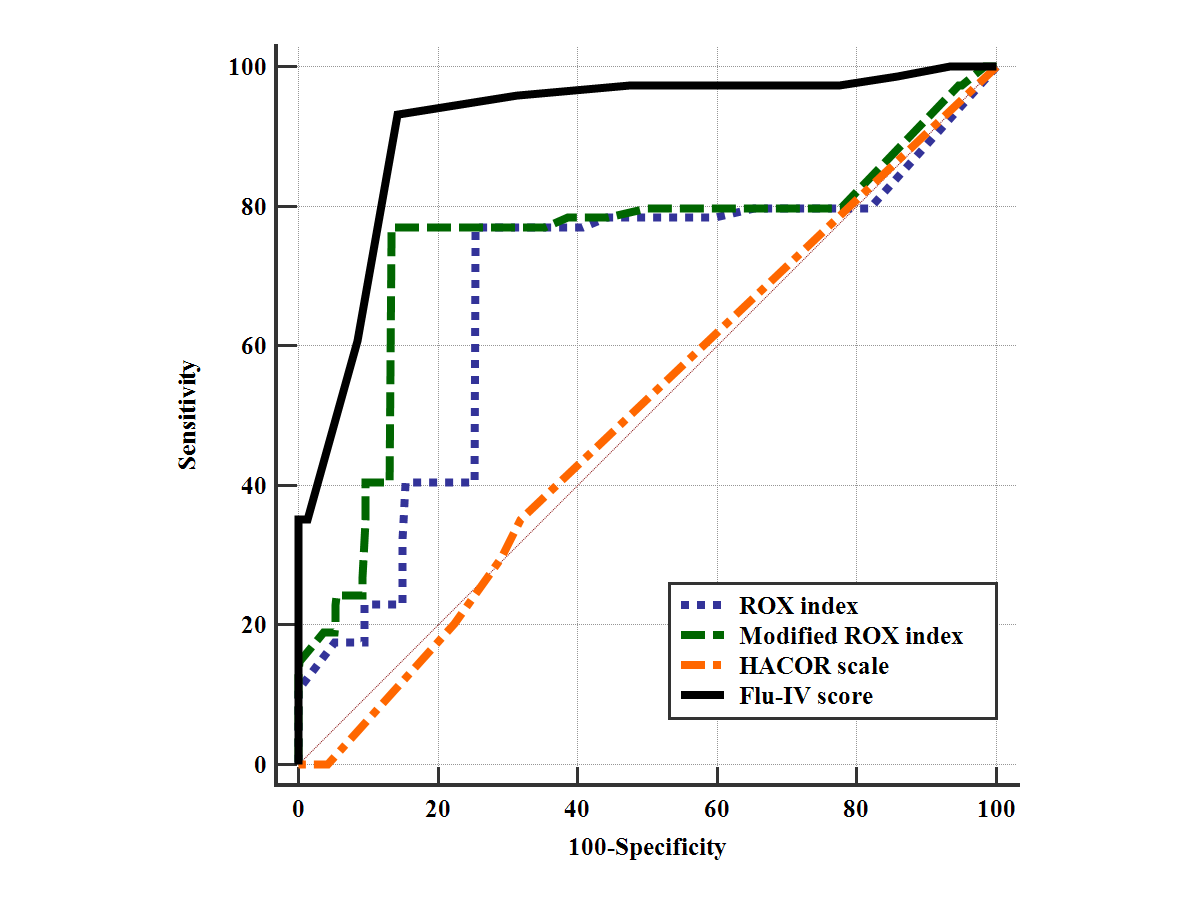


**Supplementary Fig. 1 ROCs for IMV prediction of four predictive scorings in patients from derivation cohort**

1. Hosmer-Lemeshow goodness of fit test，chi-square = 6.567， p = 0.764
2. Hosmer-Lemeshow goodness of fit test，chi-square = 3.277， p = 0.863

Supplementary Fig. 2 Calibration plots in the study cohorts for the prediction model. The dotted line shows the actual relation between observed outcomes and predicted risks; the solid line shows the smoothed relation.
